# Supplementary material for: Dissociable roles of human frontal eye fields and early visual cortex in presaccadic attention
Source: Nat Commun. 2023 Sep 4;14:5381. doi: 10.1038/s41467-023-40678-z (PMC10477327; doi:10.1038/s41467-023-40678-z)
Supplement: Supplementary file 1 — Supplementary Information [file 41467_2023_40678_MOESM1_ESM.pdf]

Supplementary Information

Dissociable roles of human frontal eye fields and early visual cortex in presaccadic attention

Nina M. Hanning[1,2*], Antonio Fernández[1,3], Marisa Carrasco[1]

[1] Department of Psychology & Center for Neural Sciences, New York University, New York, NY, USA
[2] Institut für Psychologie, Humboldt Universität zu Berlin, Berlin, Germany
[3] Department of Psychology, University of Texas at Austin, Austin, TX, USA

* corresponding author: hanning.nina@gmail.com

Supplementary Figures & References

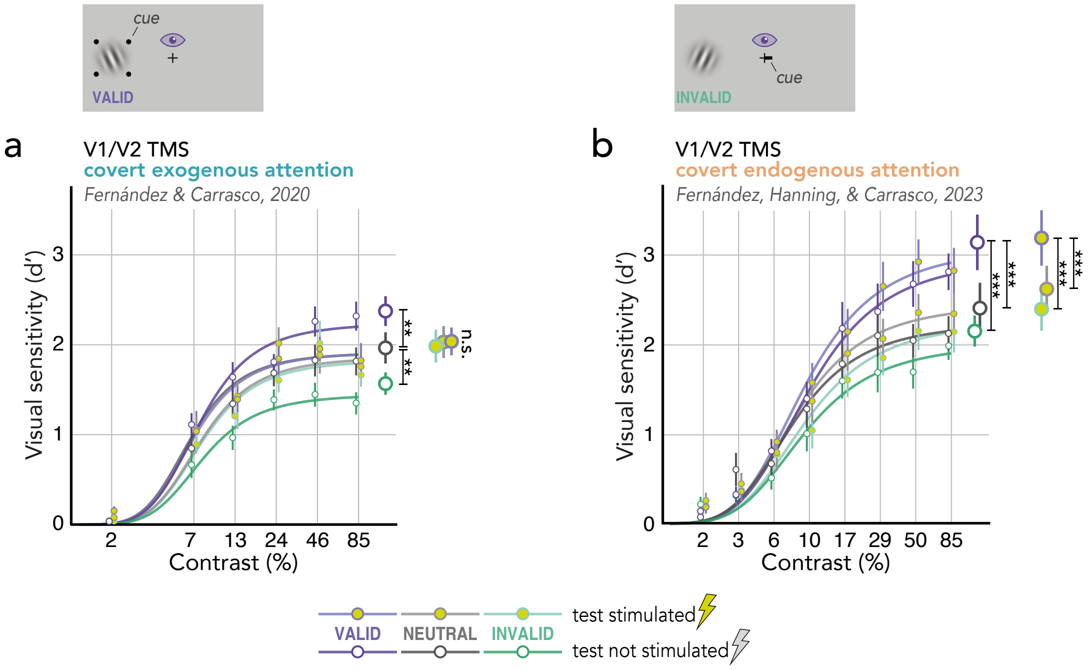

Supplementary Figure 1. The effect of V1/V2 TMS on covert attention. (**a**) Exogenous attention (n = 10, data previously published[1]). Contrast Response Functions (CRF) for *valid* trials (target at cued location; purple), *invalid* trials (target opposite cued location; green), and *neutral* trials (both locations cued; gray), measured at the stimulated region (yellow symbols) or in the symmetric, non-stimulated hemifield (white symbols). Respective group averaged parameter estimates for the upper asymptote d_{max} (based on individual observers' fits) displayed on the right. Error bars indicate ±1SEM. (**b**) Endogenous attention (n = 12, data previously published[2]). Same conventions as in (a). Source data are provided as a Source Data file. Statistical comparison (two-sided) via repeated-measures ANOVA and Bonferroni corrected posthoc t-tests; ** p<.01, *** p<.001.

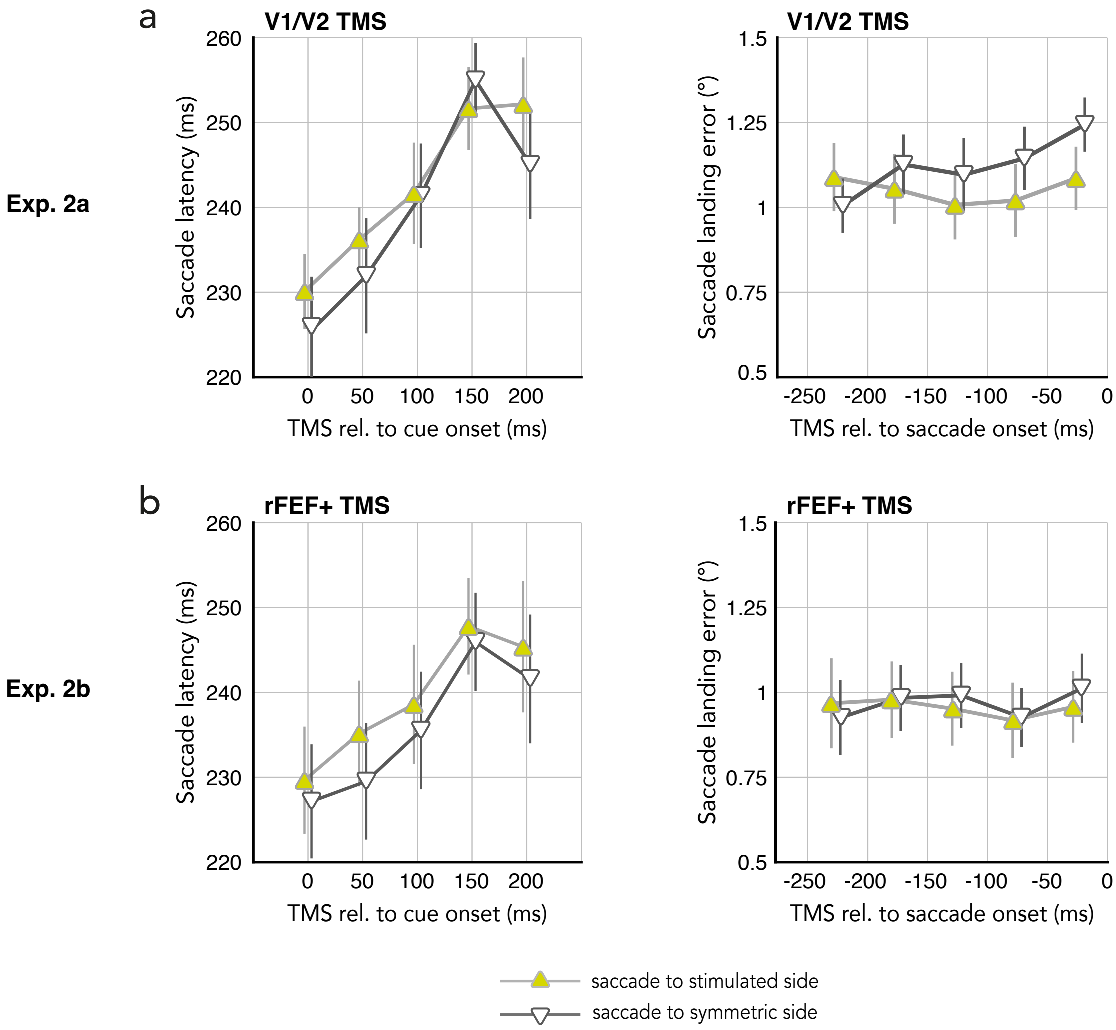

Supplementary Figure 2. Eye movement parameters. Group-averaged (mean) saccade latencies (left) and precision (landing error, computed as the distance between saccade landing position and saccade target center; right) in Experiment 2a (n = 9), V1/V2 TMS (**a**) and Experiment 2b (n = 7), rFEF+ TMS (**b**), computed based on individual participant's median measurements. Data plotted as a function of TMS pulse relative to cue onset (saccade latencies, left) or TMS pulse relative to saccade onset (landing error, right). Error bars indicate ±1SEM. TMS over both stimulation sites increased saccadic latencies (the later the TMS pulse, the later saccade onset). This pattern was not side-specific (it occurred similarly for saccades directed to the stimulated and symmetric side), and thus likely is explained by a general alerting effect of the TMS sound / sensation rather than the stimulation itself. Saccade precision was not affected by V1/V2 or rFEF+ TMS at any tested timepoint. Source data are provided as a Source Data file.

Supplementary References

1. Fernández, A., and Carrasco, M. (2020). Extinguishing Exogenous Attention via Transcranial Magnetic Stimulation. *Current Biology 30*(20), 4078–4084.
2. Fernández, A., Hanning, N.M., and Carrasco, M. (2023). Transcranial magnetic stimulation to frontal but not occipital cortex disrupts endogenous attention. *Proceedings of the national academy of sciences, 120*, e2219635120.
