# Supplementary material for: Dissociable roles of human frontal eye fields and early visual cortex in presaccadic attention
Source: Nat Commun. 2023 Sep 4;14:5381. doi: 10.1038/s41467-023-40678-z (PMC10477327; doi:10.1038/s41467-023-40678-z)
Supplement: Supplementary file 3 — Description of Additional Supplementary Files [file 41467_2023_40678_MOESM3_ESM.pdf]

File name: Supplementary Movie 1

Description: Demonstration of one trial per experimental condition (trial order: valid, invalid, neutral).
