# Supplementary material for: Dissociable roles of human frontal eye fields and early visual cortex in presaccadic attention
Source: Nat Commun. 2023 Sep 4;14:5381. doi: 10.1038/s41467-023-40678-z (PMC10477327; doi:10.1038/s41467-023-40678-z)
Supplement: Supplementary file 5 — Reporting Summary [file 41467_2023_40678_MOESM5_ESM.pdf]

nature portfolio

Corresponding author(s): Nina M. Hanning

Last updated by author(s): Jul 20, 2023

Reporting Summary

Nature Portfolio wishes to improve the reproducibility of the work that we publish. This form provides structure for consistency and transparency in reporting. For further information on Nature Portfolio policies, see our Editorial Policies and the Editorial Policy Checklist.

Statistics

For all statistical analyses, confirm that the following items are present in the figure legend, table legend, main text, or Methods section.

n/a	Confirmed	
☐	☒	The exact sample size (*n*) for each experimental group/condition, given as a discrete number and unit of measurement
☐	☒	A statement on whether measurements were taken from distinct samples or whether the same sample was measured repeatedly
☐	☒	The statistical test(s) used AND whether they are one- or two-sided<br>*Only common tests should be described solely by name; describe more complex techniques in the Methods section.*
☒	☐	A description of all covariates tested
☐	☒	A description of any assumptions or corrections, such as tests of normality and adjustment for multiple comparisons
☐	☒	A full description of the statistical parameters including central tendency (e.g. means) or other basic estimates (e.g. regression coefficient) AND variation (e.g. standard deviation) or associated estimates of uncertainty (e.g. confidence intervals)
☐	☒	For null hypothesis testing, the test statistic (e.g. *F*, *t*, *r*) with confidence intervals, effect sizes, degrees of freedom and *P* value noted<br>*Give P values as exact values whenever suitable.*
☒	☐	For Bayesian analysis, information on the choice of priors and Markov chain Monte Carlo settings
☒	☐	For hierarchical and complex designs, identification of the appropriate level for tests and full reporting of outcomes
☒	☐	Estimates of effect sizes (e.g. Cohen's *d*, Pearson's *r*), indicating how they were calculated

Our web collection on statistics for biologists contains articles on many of the points above.

Software and code

Policy information about availability of computer code

Data collection	The experimental code to present stimuli, collect behavioral responses, monitor eye movements, and control the timing of the delivered TMS pulses was written in MATLAB R2019a (MathWorks, Natick, MA, USA) using Psychtoolbox-3 (http://psychtoolbox.org) and the EyeLink toolbox (http://http://psychtoolbox.org/docs/EyelinkToolbox).
Data analysis	Behavioral and eye position data were analyzed using customized MATLAB code. To localize each observer's human homolog of the right frontal eye field (rFEF+), we mapped the right FEF+ onto their native volume using FreeSurfer FS7 (https://freesurfer.net). Analysis code to generate manuscript figures is available from the OSF database at https://osf.io/pcunw/.

For manuscripts utilizing custom algorithms or software that are central to the research but not yet described in published literature, software must be made available to editors and reviewers. We strongly encourage code deposition in a community repository (e.g. GitHub). See the Nature Portfolio guidelines for submitting code & software for further information.

Data

Policy information about availability of data

All manuscripts must include a data availability statement. This statement should provide the following information, where applicable:

- Accession codes, unique identifiers, or web links for publicly available datasets
- A description of any restrictions on data availability
- For clinical datasets or third party data, please ensure that the statement adheres to our policy

Source data are provided with this paper. Raw eye tracking and behavioral data are available from the OSF database at https://osf.io/pcunw/.

Human research participants

Policy information about studies involving human research participants and Sex and Gender in Research.

Reporting on sex and gender	Biological sex was determined by self-report.
Population characteristics	See Research sample below.
Recruitment	See Sampling strategy below.
Ethics oversight	The protocols for the study were in accordance with the safety guidelines for TMS research and approved by the University Committee on Activities Involving Human Subjects at New York University and all experimental procedures were in agreement with the Declaration of Helsinki.

Note that full information on the approval of the study protocol must also be provided in the manuscript.

Field-specific reporting

Please select the one below that is the best fit for your research. If you are not sure, read the appropriate sections before making your selection.

☐ Life sciences ☒ Behavioural & social sciences ☐ Ecological, evolutionary & environmental sciences

For a reference copy of the document with all sections, see nature.com/documents/nr-reporting-summary-flat.pdf

Behavioural & social sciences study design

All studies must disclose on these points even when the disclosure is negative.

Study description	We assessed the quantitative effect of transcranial brain stimulation on perceptual performance during saccadic eye movement preparation, using psychophysical measurements (perceptual reports via button presses) and eye movement recordings.
Research sample	Members of the New York University Community (undergraduates, masters students, PhD students, post-docs) without history of neurological or psychiatric illness and without contraindications for Transcranial Magnetic Stimulation. 10 observers (7 female; aged 22–36 years) participated in Experiment 1. 10 observers (8 of which participated in Experiment 1; 8 female; aged 21–36 years) participated in Experiment 2a, of which 7 observers (7 female; aged 22–36 years) also participated in Experiment 2b. All observers were neurologically healthy, had normal or corrected-to-normal vision, provided written informed consent, and (except for one author) were naive to the purpose of the experiment. The study assessed human vision which does not vary greatly in key characteristics such as age, gender, socioeconomic status, etc., thus this sample is representative of vision across the population.
Sampling strategy	We sampled from the NYU participant pool at convenience and availability without regard to gender or handedness. We chose a sample size in the range of previous psychophysics-TMS studies investigating presaccadic and covert attention (Fernández, A., & Carrasco, M. (2020). Extinguishing exogenous attention via transcranial magnetic stimulation. Current Biology, 30(20), 4078-4084; Fernández, A., Hanning, N. M., & Carrasco, M. (2023). Transcranial magnetic stimulation to frontal but not occipital cortex disrupts endogenous attention. Proceedings of the National Academy of Sciences, 120(10), e2219635120.; Neggers, S. F., Huijbers, W., Vrijlandt, C. M., Vlaskamp, B. N., Schutter, D. J., & Kenemans, J. L. (2007). TMS pulses on the frontal eye fields break coupling between visuospatial attention and eye movements. Journal of Neurophysiology, 98(5), 2765-2778.; Van Ettinger-Veenstra, H. M., Huijbers, W., Gutteling, T. P., Vink, M., Kenemans, J. L., & Neggers, S. F. (2009). fMRI-guided TMS on cortical eye fields: the frontal but not intraparietal eye fields regulate the coupling between visuospatial attention and eye movements. Journal of Neurophysiology, 102(6), 3469-3480.)
Data collection	Gaze position of the dominant eye was recorded using an EyeLink 1000 Desktop Mount eye tracker (SR Research, Osgoode, Ontario, Canada) at a sampling rate of 1 kHz. Manual responses (perceptual judgments) were recorded via a standard keyboard using custom MATLAB code and the Psychophysics toolbox. The experimenter was not blind to the study hypothesis. Experimental conditions were counter-balanced within each experiment (within-subject design).

Timing	Experiment 1: October 2020 – December 2020<br>Experiment 2a: January 2021 – November 2021<br>Experiment 2b: December 2021 – April 2022
Data exclusions	After preprocessing the eye position data, we included (1) trials in which no blink occurred during the trial and correct eye fixation was not maintained within a 1.75° radius centered on central fixation throughout the trial (fixation trials) or until cue onset (saccade trials); (2) eye movement trials in which the initial saccade landed within 2.0° from the required target location; (3) eye movement trials in which the test signal was presented within 100ms before saccade onset (i.e., the saccade started before test signal presentation offset or more than 100ms after test signal presentation offset).<br>Data of one observer in Experiment 2a were not included in the analysis due to an insufficient number of trials in the earliest time bin. Note that the pattern of results and reported statistical (null-)effects were not affected by this exclusion. No observers were excluded in Experiment 1 and 2b.
Non-participation	No participant dropped out, but some participants of Experiment 2a were not able to participate in Experiment 2b due to TMS contraindications.
Randomization	Within each experiment, participants performed all conditions in a counterbalanced order (within-subject design).

Reporting for specific materials, systems and methods

We require information from authors about some types of materials, experimental systems and methods used in many studies. Here, indicate whether each material, system or method listed is relevant to your study. If you are not sure if a list item applies to your research, read the appropriate section before selecting a response.

Materials & experimental systems

n/a	Involved in the study
☒	☐ Antibodies
☒	☐ Eukaryotic cell lines
☒	☐ Palaeontology and archaeology
☒	☐ Animals and other organisms
☒	☐ Clinical data
☒	☐ Dual use research of concern

Methods

n/a	Involved in the study
☒	☐ ChIP-seq
☒	☐ Flow cytometry
☒	☐ MRI-based neuroimaging
